# Dimensional model on how familial vulnerability and environmental factors impact transitional age youth psychopathology: The *Transition_psy* study

**DOI:** 10.3389/fpsyt.2023.1103030

**Published:** 2023-03-23

**Authors:** Simone Marchini, Joana Reis, Ella Ben-Shaool, Marie Delhaye, Charles Kornreich, Hélène Nicolis, Hichem Slama, Christophe Leys, Véronique Delvenne

**Affiliations:** ^1^Faculty of Medicine, Université Libre de Bruxelles (ULB), Brussels, Belgium; ^2^Department of Psychiatry for Infant, Child, Adolescent and Youth, University Hospital Brussels (HUB), Brussels, Belgium; ^3^Department of Neuropsychology and Speech Therapy, University Hospital Brussels (HUB), Brussels, Belgium; ^4^Department of Psychiatry, Brugmann University Hospital, Brussels, Belgium; ^5^Mental Health Service, Université Libre de Bruxelles (ULB), Brussels, Belgium; ^6^Faculty of Psychological Sciences and Education, Université Libre de Bruxelles (ULB), Brussels, Belgium

**Keywords:** transitional age youth, psychopathology, dimensional approach, familial vulnerability, environmental factors, quality of life, care needs

## Abstract

**Background:**

Understanding psychopathology in transitional age youth (TAY) requires a complex model, incorporating familial vulnerability and environmental factors. A trans-diagnostic and dimensional approach seems the most appropriate. *Transition_psy* study aims to assess factors playing a role in TAY psychopathology and to define predictors.

**Materials and methods:**

This article presents part of the *Transition_psy* study results, a case-control observational study. Youth aged 17 years old were recruited between June 2020 and December 2021, from both clinical [clinical population (CP) group] and non-clinical settings [non-clinical population (NCP) group]. Participants completed self-report questionnaires. The primary outcome to assess TAY psychopathology was the Youth-Self Report (YSR). We evaluated care needs with the Health of The Nation Outcome Scales For Children And Adolescents (HoNOSCA-SR) and quality of life with the World Health Organization Quality of Life – BREF (WHOQoL-BREF). Exposure factors included familial vulnerability, childhood, and present environmental factors, such as first-degree family history of psychopathology, the Childhood Trauma Questionnaire (CTQ) and the Family Assessment Device (FAD). YSR scores were compared, between groups, according to exposure factors with ANOVA and linear regression. We performed best subsets selection of multivariable analyses based on the Akaike Information Criterion. This study was registered with ClinicalTrials.gov (Identifier: NCT04333797).

**Results:**

A total of 220 TAY (CP = 106, NCP = 114) were included in the study. Participants were aged 17 years old. The majority were female (69.1%), single (96.8%), and born in Belgium (82.3%). Clinical data were all significantly different between CP and NCP groups. YSR scores were found statistically different according to group (*p* < 0.001), first-degree family history of psychopathology (*p* < 0.001), CTQ (*p* < 0.001), and FAD (*p* < 0.001). Predictive dimensional model suggested that TAY psychopathology can be predicted by group, CTQ and FAD. Significant positive correlation was found between YSR and HoNOSCA (rho = 0.81) and negative correlation between YSR and physical and psychological health (rho = −0.69 and −0.71, respectively).

**Conclusion:**

This study findings allowed to present a predictive dimensional model on TAY psychopathology, including belonging to a clinical population at transitional age, childhood trauma, and family dysfunction. Further research is needed to replicate *Transition_psy* study results in other samples. The proposed model could be used in clinical practice to improve assessment of TAY psychopathology.

## 1. Introduction

Transitional age youth (TAY), aged 16-24 years old, are a particularly at-risk population regarding mental health. Mental disorders onset before the age of 25 in 62.5% of the cases, with a peak incidence around 14.5 years old (1).

The ongoing hypothesis explaining the incidence of psychiatric disorders in TAY is multifactorial. Genetic and environmental factors, partially through epigenetics, negatively impact brain development (2). The brain maturation process presents two particularly vulnerable periods in life: (1) perinatal and early childhood, and (2) adolescence and early adulthood. During these periods, there is a greater risk that environmental factors interfere with brain maturation (2, 3).

It has already been proven that both familial vulnerability and childhood adverse events increase the risk of psychopathology (4, 5), manifested through internalizing and externalizing behaviors (6, 7). Additionally, psychopathology appears to be related to adolescent environmental factors, such as gonadal hormones, substance use, social interactions and school environment (8). It is still unclear whether these factors are a result of alterations that occurred earlier in life or strictly related to puberty (9).

All these factors contribute to the scientific and clinical complexity of TAY psychopathology and care needs. Understanding typical developmental processes remains crucial in research on the prevention of adverse life events effects on mental health (10). There is growing evidence in the literature that this complexity could be resumed in a single dimension of psychopathology, the “p” factor, measuring each individual’s liability to mental disorder, comorbidities, duration and severity of disorders. The “p” factor seems positively correlated with family history of psychiatric illness, brain function, childhood developmental history, and adult life impairment (11).

Hence, a trans-diagnostic dimensional approach seems to better allow the understanding of TAY psychopathology (12, 13). TAY psychopathology is often characterized by early clinical presentations that include non-specific or subthreshold intensity and/or frequency symptoms, and by a high incidence of comorbid disorders (14). In recent years, such trans-diagnostic clinical staging models have gained importance, by allowing a multidimensional assessment while considering illness as a dynamic continuum from its absence to its most extreme expression (13). This broader strategy to identify at-risk TAY may ultimately permit to recognize early stages of severe mental disorders, offering new management strategies tailored to the patient’s clinical stage, preventing the onset and/or progression of mental disorders (12). However, to date, there is little data on clinical dimensional characteristics involved in the development of psychopathology at the age of the transition and the identification of at-risk TAY.

**FIGURE 1:**
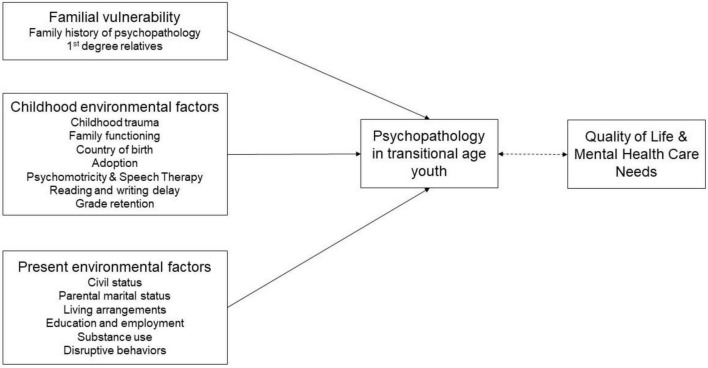
Conceptual model for the study. The single headed and continuous arrows indicate a predictive relation; the double headed and dotted arrow indicates a correlation.

The *Transition_psy* study focuses on the understanding of TAY psychopathology mainly in terms of quality of life and care needs (15). Based on the existing literature, we hypothesized that childhood trauma and family history of psychopathology were the main common factors to develop psychopathology at the transitional age (4, 5, 11). The *Transition_psy* study proposes a predictive dimensional model considering familial vulnerability, and both childhood and present environmental factors. The conceptual model of the study is presented in Figure 1. The aim of *Transition_psy* is to determine which factors play a protective or a risk role in psychopathology in the transitional age.

## 2. Materials and methods

### 2.1. Study design and setting

This article presents part of the *Transition_psy* study results. This is a case-control observational study evaluating risk and protective factors to develop psychopathology in TAY. The recruitment has been led in clinical and non-clinical settings. Clinical settings consisted in both outpatient and inpatient facilities within the urban area of Brussels: three general university hospitals and one outpatient university clinic. Non-clinical settings were both schools in the urban area of the same town and social networks. The complete protocol of *Transition_psy* study was described in a previous paper (15). This trial was registered with ClinicalTrials.gov (Identifier: NCT04333797) on 3 April 2020.

### 2.2. Recruitment and procedure

Between June 2020 and December 2021, 17 years old youth were recruited in the Brussels urban area, Belgium. Clinical sample was recruited in collaboration with the referring physician. Participants from non-clinical settings were invited to participate in the study throughout flyers and social media posts. As a compensation, they received a 20 € voucher from a multimedia shop.

To be included in the study, participants had to have sufficient fluency in French, and both parents or legal holders of parental authority and the participant had to provide informed and written consent. We excluded potential participants actively involved in another research study, those who were unable to answer the assessment tools, and patients with a progressive illness affecting short-term vital prognosis.

The clinical group was named “clinical population” (CP) because these participants were actively involved in outpatient or inpatient care, at the moment of inclusion. On the contrary, the non-clinical population was named “non-clinical population” (NCP).

At the inclusion, participants met the research assistants for a brief interview and were invited to complete the baseline assessment, consisting of self-report questionnaires available on the Research Electronic Data Capture (REDCap) platform. The time for completion was about 45 min.

**FIGURE 2:**
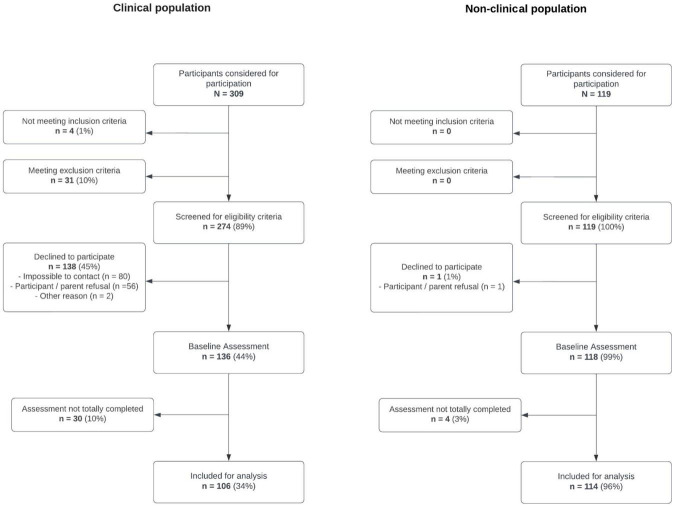
*Transition_psy* study recruitment flowchart.

Of the 428 participants considered for the study participation (CP = 309, NCP = 119), 393 were eligible to participate, among whom 254 consented to enroll in the baseline assessment. Few participants (*n* = 34) did not complete at least 50% of the assessment and they were not included in the data analysis, representing a final sample of 220 (CP = 106, NCP = 114) with a participation rate of 51.4% (see Figure 2 for more details).

### 2.3. Measures and outcomes

All the measures are self-reported standardized questionnaires, previously validated in French.

The primary outcome to assess psychopathology was the Youth Self-Report 11–18 (YSR), a specific instrument for 11–18 years old adolescents performing a detailed assessment of participants’ psychopathology (16, 17). This is a 112-item questionnaire on a three-point Likert scale (0–2). Global internalizing and externalizing problem behavior scores can be obtained. For this study analysis, we used the total scores to describe the overall TAY psychopathology (18). We also used the General Health Questionnaire-12 (GHQ) (19, 20), the 12-item version, as a screening tool for psychopathology in the sample. Each GHQ item scored on a 4-point scale (0–3); total scores range from 0 to 36 with higher scores representing greater psychopathology. This tool has been proven effective in primary care settings (21, 22).

We used two measures to evaluate care needs and quality of life, respectively: the self-rated Health of The Nation Outcome Scales For Children And Adolescents (HoNOSCA) (23, 24) and the World Health Organization Quality of Life- BREF (WHOQoL) (25, 26). The HoNOSCA (23, 24) is a 13-item instrument, scored on a five-point scale (0–4), measuring the severity of physical, personal, and social problems of children and young people with mental health problems. The total score, ranging from 0 to 52, represents the overall severity of care needs. The WHOQoL (25, 26) is a 26-item questionnaire on a five-point scale (1–5). It is possible to calculate four separate scores for each broad domain ranging from 0 to 100. The four domains of quality of life are (1) physical health, (2) psychological health, (3) social relationships, and (4) environment. These two measures were used to examine the correlation between the TAY psychopathology and their actual degree of care need and impact on quality of life.

We collected sociodemographic data, such as: sex, civil status, country of birth, parental marital status, living arrangements, enrollment in education and/or employment. Additionally, participants were asked to report clinical data, mainly related to mental health: psychiatric and/or psychological consultations, psychiatric inpatient care and psychotropic drugs use.

Exposures have been assigned to three main categories according to chronological criteria: (1) familial vulnerability, (2) childhood environmental factors, and (3) present environmental factors.

To evaluate familial vulnerability, participants were asked to provide data about mental illness history of their first-degree relatives (parents). Summarized scores could range from 0 to 2, if they had no relatives with mental illness history, one or both parents, respectively.

Childhood environmental factors were assessed using two standardized tools: the Childhood Trauma Questionnaire (CTQ) and the Family Assessment Device (FAD), together with data about history of migration, adoption, previous psychomotricity or speech therapy, grade retention at school and reading or writing delay. Concerning these two last items, the threshold age for delay was set at 8 years old (27, 28). The CTQ is a 28-item instrument measuring trauma during childhood (29, 30). Each item scores on a five-point Likert scale (1–5) and the CTQ total score ranges from 28 to 140 (31). The higher scores represent greater intensity of childhood trauma. The FAD is a 12-item tool on a four-point scale (1–4) (32, 33), with a total score from 12 to 48 (34). Higher scores represent worse levels of family functioning.

The present environmental factors considered in the study were: some of the sociodemographic variables (youth’s civil status, parental marital status, living arrangements, enrollment in education and/or employment), substance use (alcohol, tobacco, cannabis, or other drugs), and disruptive behaviors (such as stealing, vandalism, animal cruelty, assault, and battery).

### 2.4. Statistical analysis

We performed the “*a priori*” analysis to calculate the sample size with the G*Power software, version 3.1.9.7 (35). To reach a medium effect size (i.e., r = 0.3), with an α-error of 5% (two-sided) and a β-error of 80%, the required total sample size was 84 participants for correlations and 144 for linear regressions.

All statistical analyses were conducted using the software Statistical Package for the Social Sciences Version 27 (SPSS, Inc., Chicago, IL, USA).

We performed descriptive statistics to study sociodemographic and clinical characteristics in the total sample and in the two groups (CP and NCP): absolute and relative frequencies were presented for all qualitative variables; means with standard deviations were used to describe normally distributed quantitative variables. The normality of the distributions was established graphically (histogram, and normal probability plot). The frequencies in categories were compared between groups with χ^2^ test for the majority of variables; when the absolute count was less than five, we performed Fisher’s exact test. The homogeneity of variances was verified with Levene’s test, and the means of quantitative variables were compared between the two groups with independent samples T*-*test.

To compare the means of quantitative variables (GHQ and YSR) according to familial vulnerability and environmental factors between the sub-groups, we performed ANOVA for each categorical variable and linear regression for the quantitative variables (CTQ and FAD), presenting regression coefficients (b) and 95% confidence intervals. The interaction of each variable with the group was assessed in the models. For significant variables, we then performed multivariable analyses and tested all combinations of explanatory variables to choose the best model based on the Akaike information criterion (AIC). We verified the normality and homogeneity of variances of residuals with graphical representations. When appropriate, the *p*-values of post-hoc pairwise comparison tests were adjusted with Tukey’s honestly significant difference method.

Correlation between WHO-QoL, HoNOSCA, YSR, and GHQ was calculated with Spearman’s rho coefficient.

Missing data were treated with pairwise deletion. The statistical significance level was set at 0.05 (two-sided).

## 3. Results

### 3.1. Sociodemographic and clinical characteristics

A total of 220 participants, aged 17 years old, were included in the final sample. The majority of participants were female (69.1%), single (96.8%), and born in Belgium (82.3%), but there were no significant statistical differences between groups regarding sex (*p* = 0.334), civil status (*p* = 0.714), or country of birth (*p* = 0.669). There were significant statistical differences regarding parental marital status (*p* = 0.008), living arrangements (*p* = 0.009), and enrollment in education and/or employment (*p* = 0.044) between CP and NCP participants. Detailed sociodemographic characteristics of participants are presented in Table 1.

**TABLE 1:** Sociodemographic characteristics of the sample and comparisons of the groups (*N* = 220).

Variables	Total (*n* = 220)		CP (*n* = 106)		NCP (*n* = 114)		
	***n***	**%**	***n***	**%**	***n***	**%**	***p*-value**
**Sex**							
Male	66	30.0	32	30.2	34	29.8	0.334
Female	152	69.1	72	67.9	80	70.2	
Other	2	0.9	2	1.9	0	0.0	
**Civil status**							
Single	213	96.8	102	96.2	110	97.4	0.714
Cohabitant	7	3.2	4	3.8	3	2.6	
**Country of birth**							
Belgium	181	82.3	86	81.1	95	83.3	0.669
Other	39	17.7	20	18.9	19	16.7	
**Adoption**							
No	214	97.3	103	97.2	111	97.4	1.000
Yes	6	2.7	3	2.8	3	2.6	
**Parental marital status**							
Married	105	47.7	40	37.7	65	57.0	0.008**
Divorced/separated	93	42.3	56	52.8	37	32.5	
Other	22	10.0	10	9.4	12	10.5	
**Living arrangements**							
Family	202	91.8	92	86.8	110	96.5	0.009**
Other	18	8.2	14	13.2	4	3.5	
**Education and/or employment**							
Yes	207	94.1	96	90.6	111	97.4	0.044*
No	13	5.9	10	9.4	3	2.6	

*p < 0.05 and **p < 0.01. CP, clinical population; NCP, non-clinical population. Bold values are the statistically significant *p*-values.

Table 2 shows clinical data in the total sample and between groups. CP participants had significantly greater rates of psychiatric and/or psychological consultation (*p* < 0.001), inpatient care (*p* < 0.001), and psychotropic drug use (*p* < 0.001), comparing to NCP participants. CP participants had significantly higher GHQ (*p* < 0.001) and YSR (*p* < 0.001) scores than NCP participants. CP participants also presented higher HoNOSCA scores (*p* < 0.001) and lower WHOQoL scores, in all four domains (*p* < 0.001).

**TABLE 2:** Comparison of clinical data by groups (*N* = 220).

Variables	Total (*n* = 220)		CP (*n* = 106)		NCP (*n* = 114)		
	***n***	**%**	***n***	**%**	***n***	**%**	***p*-value**
**Psychiatric and/or psychological consultation**							
Yes	150	68	104	98.1	46	40.4	<0.001***
No	70	32	2	1.9	68	59.6	
**Inpatient care**							
Yes	49	22	46	43.4	3	2.6	<0.001***
No	171	78	60	56.6	111	97.4	
**Psychotropic drug**							
Yes	40	18	39	36.8	1	0.9	<0.001***
No	180	82	67	63.2	113	99.1	
	**Mean**	**SD**	**Mean**	**SD**	**Mean**	**SD**	***p*-value**
GHQ	17.05	8.24	20.81	8.13	13.55	6.70	<0.001***
YSR	61.20	10.17	66.52	8.95	56.25	8.64	<0.001***
**WHOQoL**							
Domain 1	66.26	18.59	56.44	17.91	75.39	14.04	<0.001***
Domain 2	53.44	21.71	40.53	19.30	65.45	16.29	<0.001***
Domain 3	62.60	19.15	57.43	19.84	67.39	17.23	<0.001***
Domain 4	72.74	17.15	67.58	17.97	77.53	14.89	<0.001***
HoNOSCA	17.92	10.21	24.20	9.02	12.09	7.42	<0.001***

***p < 0.001. CP, clinical population; GHQ, General Health Questionnaire; HoNOSCA, Health of The Nation Outcome Scales For Children And Adolescents; NCP, non-clinical population; SD, standard deviation; WHO-QoL, World Health Organization Quality of Life (domains: (1) physical health, (2) psychological health, (3) social relationships, and (4) environment); YSR, Youth Self-Report. Bold values are the statistically significant *p*-values.

### 3.2. Familial vulnerability and environmental factors

We decided to present the analysis of YSR scores according to the group and familial vulnerability, childhood and present environmental factors as primary results (Table 3). Findings regarding GHQ scores according to the most significant factors are summarized in Table 4. The analyses of every factor using the GHQ scores are presented in the annex section (Supplementary Table 1).

**TABLE 3:** Youth-Self Report (YSR) scores according to group and familial vulnerability, childhood or present environmental factors.

Variables	Total (*n* = 220)		CP (*n* = 106)		NCP (*n* = 114)		*p*-value		
**Family vulnerability**	**Mean**	**SD**	**Mean**	**SD**	**Mean**	**SD**	**Variable**	**Group**	**Interaction variable-group**
**1^st^ degree family history^a^**									
None	58.65	9.55	64.67	8.67	55.23	8.28	0.022*	<0.001***	0.681
One parent	66.53	8.59	68.25	8.24	58.50	5.09			
Both parents	66.69	12.01	68.63	10.41	63.60	14.98			
	**b [95% CI]**						***p*-value**		
**Childhood environmental factors**	**Variable**		**Group**		**Interaction variable-group**		**Variable**	**Group**	**Interaction variable-group**
CTQ^a^	0.44 [0.22; 0.37]		–0.38 [–4.99; –2.80]		0.12 [0.01; 0.16]		<0.001***	<0.001***	0.025*
FAD^a^	0.36 [0.31; 0.58]		–0.42 [–5.38; –3.16]		0.04 [–0.08; –0.19]		<0.001***	<0.001***	0.430
	**Mean**	**SD**	**Mean**	**SD**	**Mean**	**SD**	**Variable**	**Group**	**Interaction variable-group**
**Country of birth**									
Belgium	61.03	10.32	66.64	8.78	55.96	8.54	0.715	<0.001***	0.438
Other	61.97	10.17	66.00	9.87	57.74	9.21			
**Adoption**									
Yes	61.83	8.40	67.67	4.16	56.00	7.55	0.900	0.003**	0.844
No	61.18	10.22	66.49	9.06	56.26	8.69			
**Psychomotricity**									
Yes	63.65	8.90	66.55	6.09	58.33	11.26	0.633	<0.001***	0.642
No	61.00	10.26	66.52	9.25	56.14	8.52			
**Speech therapy**									
Yes	62.61	10.33	67.61	7.71	54.35	8.74	0.798	<0.001***	0.134
No	60.66	10.08	65.91	9.58	56.74	8.59			
**Reading delay^b^**									
Yes	62.94	10.84	68.33	9.73	57.56	9.46	0.453	<0.001***	0.899
No	60.97	10.07	66.43	8.78	56.20	8.62			
**Writing delay^b^**									
Yes	58.50	11.50	67.67	10.07	54.57	10.23	0.891	<0.001***	0.642
No	61.31	10.06	66.66	8.78	56.43	8.58			
**Grade retention^b^**									
Never	59.23	10.16	67.23	9.23	55.04	7.89	0.409	<0.001***	0.059
Once	63.65	9.31	65.78	8.55	60.16	9.63			
Twice or more	64.69	10.31	66.48	9.45	57.20	11.45			
**Present environmental factors**	**Mean**	**SD**	**Mean**	**SD**	**Mean**	**SD**	**Variable**	**Group**	**Interaction variable-group**
**Civil status^b^**									
Single	61.24	10.25	66.58	9.07	56.34	8.73	0.473	0.001**	0.797
Cohabitant	59.86	7.56	65.00	5.48	53.00	1.73			
**Parental marital status^b^**									
Married	59.00	10.09	65.67	9.19	54.89	8.31	0.027*	<0.001***	0.658
Divorced/separated	64.23	9.90	67.68	8.72	59.00	9.38			
Other	58.91	8.58	63.40	9.08	55.17	6.28			
**Living arrangements^b^**									
Family	61.21	10.26	67.08	8.76	56.31	8.69	0.26	<0.001***	0.604
Other	61.06	9.74	62.86	9.70	54.75	7.85			
**Education and/or employment^b^**									
Yes	61.12	10.30	66.67	9.19	56.32	8.70	0.514	<0.001***	0.900
No	62.54	7.77	65.10	6.39	54.00	6.08			
**Alcohol^b^**									
Yes	61.69	9.70	65.15	8.63	56.40	7.38	0.284	<0.001***	0.446
No	60.42	10.88	67.38	9.38	56.02	10.42			
**Tobacco^b^**									
Yes	63.77	10.17	68.83	8.12	56.77	8.50	0.117	<0.001***	0.286
No	60.19	10.02	65.33	9.18	56.10	8.72			
**Cannabis^b^**									
Yes	63.53	9.67	67.54	8.71	57.28	7.64	0.274	<0.001***	0.911
No	60.24	10.24	65.93	9.10	55.97	8.91			
**Other drug^b^**									
Yes	65.43	7.37	67.17	6.31	55.00	0.00	0.952	0.021*	0.839
No	61.06	10.23	66.48	9.11	56.27	8.67			
**Disruptive behaviors^b^**									
Yes	65.98	9.26	70.28	7.43	58.19	6.95	0.014*	<0.001***	0.331
No	59.97	10.05	65.10	9.11	55.94	8.87			

^a^Data is missing for 24 participants (16 CP and 8 NCP); ^b^Data is missing for 9 participants (7 CP and 2 NCP); *p < 0.05; **p < 0.01; and ***p < 0.001. CTQ, Childhood Trauma Questionnaire; FAD, Family Assessment Device; CP, clinical population; NCP, non-clinical population; SD, standard deviation; YSR, Youth Self-Report. Bold values are the statistically significant *p*-values.

**TABLE 4:** Summary of the GHQ scores according to most significant factors.

Variables	Total (*n* = 196)		CP (*n* = 90)		NCP (*n* = 106)		*p*-value		
**Family vulnerability**	**Mean**	**SD**	**Mean**	**SD**	**Mean**	**SD**	**Variable**	**Group**	**Interaction variable-group**
**1^st^ degree family history**									
None	15.38	7.78	18.78	8.58	13.44	6.59	0.028*	0.001**	0.743
One parent	21.18	8.54	22.61	7.97	14.50	8.60			
Both parents	22.31	7.78	24.63	6.89	18.60	8.14			
	**b [95% CI]**						***p*-value**		
**Childhood environmental factors**	**Variable**		**Group**		**Interaction variable-group**		**Variable**	**Group**	**Interaction variable-group**
CTQ	0.24 [063; 0.020]		–0.36 [–3.97; –1.94]		0.02 [–0.06; 0.8]		<0.001***	<0.001***	0.782
FAD	0.26 [0.14; 0.38]		–0.37 [–3.99; –2.02]		–0.32 [–0.54; 0.59]		<0.001***	<0.001***	0.592
**Present environmental factors**	**Mean**	**SD**	**Mean**	**SD**	**Mean**	**SD**	**Variable**	**Group**	**Interaction variable-group**
**Other drug**									
Yes	14.00	6.25	15.83	4.31	3.00	.	0.049*	0.012*	0.504
No	17.15	8.30	21.11	8.22	13.65	6.66			

*p < 0.05; **p < 0.01; and ***p < 0.001; complete results are available in the annex section. CTQ, Childhood Trauma questionnaire; FAD, Family Assessment Device; GHQ, General Health Questionnaire; CP, clinical population; NCP, non-clinical population; SD, standard deviation. Bold values are the statistically significant *p*-values.

Youth Self-Report scores were statistically different according to the group (CP and NCP), in each analyzed factors. The most significant factors to determine differences in YSR scores were the first-degree family history for psychopathology (*p* = 0.022), CTQ scores (*p* < 0.001), FAD scores (<0.001), parental marital status (*p* = 0.027), and disruptive behaviors (*p* = 0.014). Results on the interaction between the group and each factor did not show a significant difference, except for CTQ scores (Table 3). Regarding first-degree family history for psychopathology, post-hoc pairwise comparison tests showed a significant difference between participants without family history and those with one or both parents with a mental disorder (*p* < 0.001 and *p* = 0.004 accordingly), but no statistical difference between these last two categories (*p* = 0.998).

We found similar findings on GHQ scores in both group and factors. The most significant factors to determine differences in GHQ scores were the first-degree family history for psychopathology (*p* = 0.028), CTQ scores (*p* < 0.001), FAD scores (<0.001), and other drugs use (*p* = 0.049) (Table 4).

### 3.3. Predictive dimensional models

We studied the most predictive models on YSR scores with selection of the most significant factors identified in the previous logistic regressions. The two models with the smallest AIC were selected. *Model 1* included the following variables: group, CTQ, FAD, and first-degree family history of psychopathology (AIC = 790.13). In *model 2*, the variable first-degree family history of psychopathology was excluded (AIC = 790.17). The significance of each predictor in the predictive dimensional models is shown in Table 5.

**TABLE 5:** Best model selection: significance of the predictors on YSR.

	Variables	AIC	*p*-value	Model coefficients	95% confidence interval
*Model 1*	Group	790.13	<0.001***	–0.377	(–9.997, –5.340)
	CTQ		<0.001***	0.284	(0.094, 0.310)
	FAD		0.044*	0.152	(0.005, 0.377)
	1^st^ degree family history		0.159	0.084	(–0.580, 3.507)
*Model 2*	Group	790.17	<0.001***	–0.383	(–9.994, –5.617)
	CTQ		<0.001***	0.305	(0.107, 0.310)
	FAD		0.047*	0.149	(0.003, 0.365)

*p < 0.05 and ***p < 0.001. AIC, Akaike Information Criterion; CTQ, Childhood Trauma Questionnaire; FAD, Family Assessment Device; YSR, Youth Self-Report. Bold values are the statistically significant *p*-values.

Finding about the most predictive two models for GHQ scores are presented in the annex section (Supplementary Table 2). Group, FAD, and first-degree family history of psychopathology are included in both models, whereas CTQ is only present in the second best model.

### 3.4. Quality of life and care needs

**FIGURE 3:**
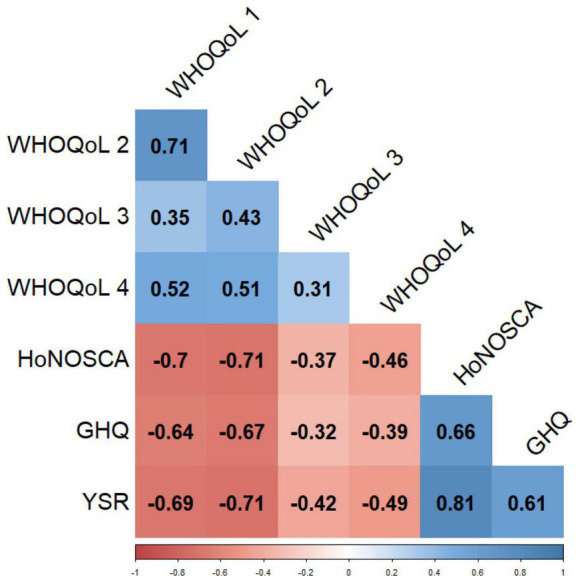
Correlation matrix between WHO-QoL, HoNOSCA, GHQ, and YSR. Correlation coefficient, method = Spearman’s rho; all coefficients are bold because they are statistically significant (*p* < 0.05). GHQ, General Health Questionnaire; HoNOSCA, Health of The Nation Outcome Scales For Children And Adolescents; WHO-QoL, World Health Organization Quality of Life (domains: (1) physical health, (2) psychological health, (3) social relationships, and (4) environment); YSR, Youth Self-Report.

The correlation matrix (Figure 3) assesses the strength and the direction of the relationship between the four domains of WHOQoL, HoNOSCA, GHQ, and YSR. Each correlation coefficient was statistically significant (*p* < 0.05). The higher positive correlation coefficient (rho = 0.81) was found in the relationship between YSR and HoNOSCA. GHQ and HoNOSCA are also positively correlated, but with a lower coefficient (rho = 0.66). Similar results were found in GHQ and YSR (rho = 0.61). GHQ, YSR, and HoNOSCA correlate negatively with all four domains of WHOQoL. However, the correlations with the highest coefficients are found between the first two domains of WHOQoL (1 = physical health; 2 = psychological health) and the other measures scores: GHQ (rho = −0.64 and −0.67, respectively), YSR (rho = −0.69 and −0.71, respectively), and HoNOSCA (rho = −0.70 and −0.71, respectively). The four domains of WHOQoL are all positively correlated among them; even if statistically significant, these correlations do not appear very strong. The highest one is the correlation between domains 1 and 2 (rho = 0.71).

## 4. Discussion

This paper presents part of the *Transition_psy* study results. It consists of a case-control observational study aiming to model the mechanisms involved in the TAY psychopathology.

### 4.1. Sociodemographic and clinical characteristics

Some sociodemographic characteristics were are significantly related to the groups. Our findings show that CP participants have more frequently divorced or separated parents which is consistent with previous literature (36, 37). The differences observed in living arrangement conditions, on the contrary, is possibly related to the recruitment strategy of the *Transition_psy* study, as the clinical facilities collaborated with Youth Aid Residential Services, resulting in greater rates of CP youth not living with their families. Enrollment in education and/or employment is lower in the CP group. According to the Belgian Superior Health Council (38), psychopathology is associated with a higher risk for school drop-out (39), and reduced work activity (40).

The statistical differences in clinical characteristics confirm that CP and NCP groups properly represent the clinical and non-clinical populations that we aimed to target in this study. Both psychopathology scores (GHQ and YSR) are relevant to distinguish between CP and NCP participants; these findings are consistent with previous research (41–43). Concerning quality of life, WHO-QoL scores in the CP population are significantly lower than in a reference healthy population (25). In particular, psychological health scores (Domain 2) in the total sample and CP group are lower than the means in the reference healthy population, whereas the environment scores (Domain 4) are higher in this study sample (25). Milestone European study showed that HoNOSCA is an appropriate instrument to assess the severity of mental health problems in TAY (44), which is consistent with our study sample.

However, part of the NCP participants already had previously had psychiatric and/or psychological consultation (40%), and a small portion of them already have received inpatient care in psychiatric units or psychotropic treatment. If these results could be partially explained by epidemiological data on mental health care needs in the general population (45), we should also highlight three additional factors. First, mental health literacy appears to have improved in youth over the last years (46), resulting in higher psychological or psychiatric consultation rates that might not be related to a general increase in psychopathology nor in mental health care needs. Second, we acknowledge that data were collected mostly during the COVID-19 pandemic. It has already been proven that mental disorders increased during the pandemic, particularly in youth (47). Last, since NCP participants volunteered to participate in this research, they could be more likely to be concerned by a past or present experience of psychopathology.

Participation rates were quite different according to the two groups (CP and NCP). Firstly, the recruitment procedure was different in CP and NCP groups. Since CP youth were enrolled in the study in collaboration with their clinician, many of them (45%) declined to participate. On the contrary, NCP youth were asked to express their interest to participate, spontaneously, and they received compensation; as consequence, they were less susceptible to decline the participation to the study. Secondly, we hypothesize that in the CP group, the intensity of the psychopathology might have discouraged participation, which lead to higher rates of refusal and impossibility to reach out the participant by phone.

### 4.2. Familial vulnerability and environmental factors

We explored the role of several factors on TAY psychopathology, which have been grouped into three main categories: (1) familial vulnerability, (2) childhood environmental factors, and (3) present environmental factors. Due the recruitment selection of participants, the two groups differed significantly in terms of psychopathology. The interaction of each factor and the group was explored.

Youth psychopathology appeared significantly different according to the presence of parental history of psychopathology, in both CP and NCP groups. However, the effect of one or both parents with history of psychopathology was not cumulative, the only significant effect on TAY psychopathology was whether at least one parent presented a positive history of mental disorder or not. It is already known that parental mental illness represents a double burden for children and adolescents in both genetic transmission (4, 48) and family-related factors, such as a worse family environment and interaction between parents and children (49). Our study findings on familial vulnerability support the link between genetics first-degree familial mental health disorders and TAY psychopathology.

All types of childhood trauma have been proven to be a common matrix in the emergence of non-specific psychopathology, playing a crucial role in the factor “p” model (11). Our study findings are consistent with the previous literature. Childhood trauma (CTQ) was statistically correlated with TAY psychopathology, and the interaction with the group was significant. This leads us to suggest that childhood trauma might trigger to seek for help at the transitional age. Further research in other TAY samples should be performed to verify this hypothesis.

Family environment plays a mediating role in the relationship between stressors and children and adolescents’ psychopathology (50, 51), but the impact on youth still needs to be established. In our TAY sample, family functioning appeared to be significantly correlated with psychopathology. We can affirm that these findings about family functioning in TAY provide consistent evidence about the relation with youth psychopathology.

Among present environmental factors, only parental marital status and disruptive behaviors were found to be significant. As discussed above, parental marital status was statistically different according to the group. Our TAY sample psychopathology appeared to correlate significantly with this factor, as is the case for children (37). The main hypothesis explaining the correlation between psychopathology and disruptive behaviors seems to be the possible redundancy of this variable with two YSR sub-scales (rule-breaking behavior, and aggressive behavior) (16). However, we should point out that total scores for psychopathology were significantly correlated to disruptive behaviors in our study sample.

The results on substance use should be discussed. None of the assessed substance was significant to TAY psychopathology. This finding could be explained in two different ways. On one hand, we could hypothesize that substance use does not intervene in the emergence of psychopathology, it is rather a consequence of psychopathology as a subsequent manifestation. On the other hand, in occidental societies, substance use is increasing in youth, and the age of first drug consumption is lower than it was in the past, and this is certainly the case in Belgium (52). Hence, substance use in youth might be not uniquely correlated to psychopathology, but also connected to societal trends.

Similar considerations can be pointed out about enrollment in education and/or employment. However, in this case, the causative role of psychopathology on school drop-out and reduced work activity has already been recognized (39, 40).

### 4.3. Predictive dimensional models

The best subsets selection aimed to present dimensional models for the most predictive outcomes on TAY psychopathology. Results on YSR identified the three best predictors in youth: group, childhood trauma (CTQ), and family functioning (FAD). Family functioning was already correlated with youth psychopathology in urban areas, particularly regarding internalizing symptoms (53). Recent studies showed a positive correlation between adverse childhood experiences and neurodevelopmental disorders in children, leading to a greater risk of poor health outcomes in childhood and adolescence through the mediation of maladaptive stress calibration (54–56). Thus, the presence of childhood trauma and poor family functioning in strong association with the psychopathology in our TAY sample seems consistent with these models. These findings also need to be interpreted in light of some CTQ specific characteristics. The majority of items in the CTQ are related to past experiences with family and parents (29, 30), consequently concordant results between CTQ and FAD seem consistent with previous studies in University students (57).

The first-degree family history of psychopathology, even if significant in the linear regression, appeared to be redundant to predict TAY psychopathology. This might be related to different hypotheses. In particular, we found an already proven correlation between maternal mood disorder, youth comorbidity, and worse family functioning among bipolar youth (58). Additionally, high parental stress is a major risk factor for childhood maltreatment. Childhood maltreatment has been proven to worsen psychopathology, in particular symptoms related to neurodevelopmental conditions (54, 59), but also to cause psychopathology if combined with other genetic and environmental risk factors (60). For these reasons, we highlight that childhood trauma and poor family functioning might be strong enough explanatory factors associated with belonging to a clinical population.

The influence of genetics on psychiatric disorders (4, 48) is far from being rejected with these findings on familial vulnerability. However, dimensional models showed that, in clinical practice, assessment of childhood trauma and family functioning in the clinical population might be more efficient in the prediction of psychopathology in TAY.

The most predictive dimensional models on GHQ showed three similar significant factors: group, family functioning (FAD), and first-degree family history of psychopathology. These findings are essential for the screening of TAY psychopathology in primary care settings. General practitioners and other first-line health professionals might benefit from this model to efficiently screen psychopathology in youth and, if needed, refer to specific psychiatric care.

### 4.4. Quality of life and care needs

The positive and strong correlation between YSR and HoNOSCA confirms that as TAY psychopathology increases, care needs are greater. HoNOSCA has already been proven to be a cheap and efficient tool to monitor care needs in youth at the transition boundary (61). This instrument should be considered in clinical practice because of its specificity to correlate with psychopathology in detail. The correlation between GHQ and HoNOSCA is positive and significant, but less strong than the correlation between YSR and HoNOSCA. These findings are consistent with the fact that GHQ is a more sensitive tool to screen psychopathology, when compared to YSR, but may be less specific to determine care needs in depth. As already discussed above, GHQ finds its interest in primary care. In general, these results about positive correlation between TAY psychopathology and care needs support the encouraging trend of mental health literacy in youth (46), which is the best strategy to improve early intervention (62, 63).

Youth psychopathology and all quality of life domains are negatively correlated in the *Transition_psy* study sample. In particular, greater correlations are found in physical and psychological health, meaning that these two domains are more related to youth psychopathology in our study population. In the clinical sample of the Milestone European study, the most impacted domains were psychological health and social relationships, even if not significant in the comparison between usual care and managed transition (61).

Concerning quality of life itself, our findings show that all four domains correlate positively among them in a significant way. TAY’s physical and psychological health positively and strongly correlates. Environment moderately correlates with both physical and psychological health, whereas the correlation between the social relationships and the other three domains is not very strong. To date, few studies are available in the literature showing WHO-QoL inter-domain correlation in TAY. One of these was conducted in war-affected youth in Sierra Leone; these findings show lower positive correlation between physical health with psychological health and environment, whereas the correlation between environment and psychological health seems more similar (64). Further research should be led to test more detailed hypotheses on this subject.

### 4.5. Limitations

Certain limitations in this study should be further taken into account, mainly the differences about the CP and NCP groups and the impact of COVID-19 pandemics on youth mental health.

Participant groups (CP or NCP) were selected according to the participants’ active involvement in inpatient and/or outpatient mental health care, in the recruitment setting. However, all data were collected *via* self-report questionnaires, resulting in the absence of clinician and/or parents information. Among the CP population, a small proposition of participants did not self-reported being involved in mental health care even if they were recruited in one of the clinical settings. This example illustrates that information bias is a key problem in the assessment of research study designs and a distortion of results must always be considered (65).

Selection bias must also be taken into account in this study. A considerable proportion of NCP youth declared involvement in inpatient or outpatient mental health care or psychotropic drug use previously to the recruitment phase. Even though they were not actively seeking mental health care, their previous life experiences may have caused a distortion in results, probably by reducing differences between the two groups. Additionally, a selective refusal to participate in the study was perhaps observed. Participants needed to be motivated to answer questions about their mental health status and psychological vulnerabilities. It is possible that some responders dropped out of the study for two opposite reasons: either their psychological discomfort was too high to participate in a mental health study (most probably in the CP group) or, on the contrary, they did not feel mental health was a priority during the considered period (particularly among the NCP group). Also, recruitment strategies between the two groups were slightly different as described in the methods section (15, 66).

Additionally, data were collected between June 2020 and December 2021, mostly during the COVID-19 pandemic phase. It has already been shown that mental health problems urged during this period, particularly in adolescents and young adults (47). The impacts of the COVID-19 pandemic on mental health research are not yet properly acknowledged but this element must be considered in our study.

## 5. Conclusion

A complex model, including familial vulnerability and environmental factors, is needed to understand the emergence of psychopathology in TAY. To date, the trans-diagnostic and dimensional approach seems to be the most appropriate one.

With this study, we propose a predictive dimensional model on TAY psychopathology that includes belonging to a clinical group at the transitional age, childhood trauma, and family functioning. To date, few studies have been conducted on how to predict psychopathology in youth. Further research is needed to replicate these findings and to study other factors’ role in TAY psychopathology.

In terms of implications in clinical practice, early and specific assessment of the emergent psychopathology in TAY is crucial to allow early intervention and to improve lifelong prognostic. The proposed predictive dimensional model might be implemented in clinical practice to alert professionals about the presence of psychopathology in TAY.

Additionally, HoNOSCA seems an efficient tool to establish care needs in psychiatric settings at the boundary between Child and Adolescent Mental Health Services and Adult Mental Health Services. GHQ could be used as a consistent screening tool of psychopathology in youth in primary care settings.

## Data availability statement

The raw data supporting the conclusions of this article will be made available by the authors, without undue reservation.

## Ethics statement

The studies involving human participants were reviewed and approved by IRBs of Queen Fabiola Children’s University Hospital, Brugmann University Hospital, and Erasmus Hospital. The study was conducted in accordance with the International Conference on Harmonization (ICH) for good clinical practice (GCP). Written informed consent to participate in this study was provided by the participants’ legal guardian/next of kin.

## Author contributions

VD was the chief investigator who conceptualized the study and wrote the grant funding proposition, together with MD, HN, CK, and JR. SM, JR, and EB-S collected and analyzed the research data and prepared the first draft and subsequent versions of this manuscript. SM and JR were the research assistants and wrote the ethical approval documents. CL and HS contributed to the statistical analysis and results interpretation. VD, HN, MD, CK, CL, and HS revised the manuscript. All authors reviewed and approved the submitted version of this manuscript.
