# Supplementary material for: Dimensional model on how familial vulnerability and environmental factors impact transitional age youth psychopathology: The Transition_psy study
Source: Front Psychiatry. 2023 Mar 23;14:1103030. doi: 10.3389/fpsyt.2023.1103030 (PMC10076738; doi:10.3389/fpsyt.2023.1103030)
Supplement: Supplementary file 1 [file Table_1.pdf]

Supplementary Material

Supplementary Table 1 – GHQ scores according to group and familial vulnerability, childhood or present environmental factors

Variables	Total (n = 220)		CP (n = 106)		NCP (n = 114)		p-value		
Family vulnerability	**Mean**	**SD**	**Mean**	**SD**	**Mean**	**SD**	**Variable**	**Group**	**Interaction Variable-group**
1st degree family history [a]									
None	15.38	7.78	18.78	8.58	13.44	6.59	***0.028****	***0.001*****	0.743
One parent	21.18	8.54	22.61	7.97	14.50	8.60			
Both parents	22.31	7.78	24.63	6.89	18.60	8.14			
	**b [95% CI]**						**p-value**		
Childhood environmental factors	**Variable**		**Group**		**Interaction Variable-group**		**Variable**	**Group**	**Interaction Variable-group**
CTQ [a]	0.24 [063 ; .020]		-0.36 [-3.97 ; -1.94]		0.02 [-0.06 ; 0.8]		***<0.001******	***<0.001******	0.782
FAD [a]	0.26 [0.14 ; 0.38]		-0.37 [-3.99 ; -2.02]		-0.32 [-0.54 ; 0.59]		***<0.001******	***<0.001******	0.592
	**Mean**	**SD**	**Mean**	**SD**	**Mean**	**SD**	**Variable**	**Group**	**Interaction Variable-group**
Country of birth									
Belgium	16.86	8.16	20.60	8.11	13.46	6.60	0.536	***<0.001******	0.832
Other	17.95	8.71	21.70	8.36	14.00	7.38			
Adoption									
Yes	18.17	6.21	21.67	7.02	14.67	3.22	0.743	***0.022****	0.966

No	17.02	8.31	20.79	8.19	13.52	6.78			
Psychomotricity									
Yes	20.06	8.64	23.09	7.02	14.50	9.09	0.367	***<0.001****	0.694
No	16.80	8.19	20.55	8.24	13.50	6.60			
Speech therapy	18.67	8.83	22.21	8.21	12.83	6.48			
Yes	16.43	7.96	20.03	8.04	13.74	6.78	0.580	***<0.001****	0.179
No									
Reading delay [b]									
Yes	17.28	9.35	18.00	10.21	16.56	8.96	0.954	***0.013****	0.090
No	16.91	8.18	21.02	7.98	13.32	6.52			
Writing delay [b]									
Yes	15.10	8.09	17.00	10.15	14.29	7.83	0.538	0.055	0.368
No	17.10	8.32	21.01	8.18	13.53	6.72			
Grade retention [b]									
Never	16.12	8.04	21.91	7.56	13.08	6.50	0.914	***<0.001****	0.115
Once	18.24	9.11	20.66	9.38	14.28	7.18			
Twice or more	18.62	6.42	18.81	6.40	17.80	7.19			
Present environmental factors	**Mean**	**SD**	**Mean**	**SD**	**Mean**	**SD**	**Variable**	**Group**	**Interaction Variable-group**
Civil status [b]									
Single	17	8.28	20.92	8.15	13.41	6.62	0.643	0.259	0.140
Cohabitant	18.43	7.70	18.00	8.08	19.00	8.89			
Parental marital status [b]									
Married	16.39	8.05	21.18	7.96	13.45	6.60	0.279	***<0.001****	0.797
Divorced/separated	18.39	8.25	20.91	8.22	14.57	6.76			
Other	14.55	8.61	18.80	8.82	11.00	6.96			
Living arrangements [b]									
Family	17.24	8.36	21.62	7.98	13.57	6.78	0.119	***0.014****	0.196
Other	14.94	6.72	15.50	7.24	13.00	4.69			
Education and/or employment [b]									
Yes	17.15	8.30	21.22	8.08	13.64	6.77	0.127	***0.005****	0.839
No	15.38	7.45	16.90	7.89	10.33	1.53			

Alcohol [b]									
Yes	17.01	8.21	20.52	8.27	13.76	6.71	0.917	***<0.001******	0.537
No	17.11	8.36	21.27	7.98	13.23	6.75			
Tobacco [b]									
Yes	17.40	8.03	20.28	7.61	13.42	6.91	0.666	***<0.001******	0.777
No	16.91	8.36	21.09	8.42	13.59	6.68			
Cannabis [b]									
Yes	17.22	8.24	20.23	8.04	12.52	6.19	0.321	***<0.001******	0.858
No	16.98	8.28	21.15	8.22	13.84	6.84			
Other drug [b]									
Yes	14.00	6.25	15.83	4.31	3.00	.	***0.049****	***0.012****	0.504
No	17.15	8.30	21.11	8.22	13.65	6.66			
Disruptive behaviors [b]									
Yes	16.58	8.07	19.28	7.71	11.69	6.36	0.097	***<0.001******	0.983
No	17.17	8.31	21.39	8.25	13.86	6.74			

Legend: [a] Data is missing for 24 participants (16 CP and 8 NCP) ; [b] Data is missing for 9 participants (7 CP and 2 NCP); * p<0.05; ** p<0.01; *** p<0.001.
Abbreviations: CP, clinical population; CTQ, Childhood Trauma questionnaire; FAD, Family Assessment Device; GHQ, General Health Questionnaire; NCP, non-clinical population; SD, standard deviation.

Supplementary Table 2 – Best model selection: significance of the predictors on GHQ

	**Variables**	**AIC**	**p-value**	**Model Coefficient**	**95% confidence interval**
Model 1	Group<br>FAD<br>1st degree family history	766.69	**<0.001*****<br>**<0.001*****<br>**0.038***	-0.295<br>0.253<br>0.140	(-7.043, -2.709)<br>(0.125, 0.393)<br>(0.110, 3.881)
Model 2	Group<br>FAD<br>1st degree family history<br>CTQ	768.31	**<0.001*****<br>**0.012***<br>0.052<br>0.543	-0.288<br>0.220<br>0.133<br>0.054	(-6.968, -2.567)<br>(0.050, 0.401)<br>(-0.035, 3.827)<br>(-0.071, 0.134)

Legend: * p<0.05; *** p<0.001
Abbreviations: AIC, Akaike Information Criterion; CTQ, Childhood Trauma Questionnaire; FAD, Family Assessment Device; GHQ, General Health Questionnaire.
